# Exploring the Unique Properties and Superior Schwann Cell Guiding Abilities of Spider Egg Sac Silk

**DOI:** 10.1021/acsabm.4c01587

**Published:** 2025-01-17

**Authors:** Karolina Peter, Sarah Stadlmayr, Aida Naghilou, Leon Ploszczanski, Manuel Hofmann, Christian Riekel, Jiliang Liu, Manfred Burghammer, Claudia Gusenbauer, Johannes Konnerth, Hannes C. Schniepp, Harald Rennhofer, Gerhard Sinn, Christine Radtke, Helga C. Lichtenegger

**Affiliations:** † Institute of Physics and Materials Science, Department of Natural Sciences and Sustainable Ressources, BOKU University, Peter Jordan-Straß 82, 1190 Vienna, Austria; ‡ Department of Plastic, Reconstructive and Aesthetic Surgery, Medical University of Vienna, Währinger Gürtel 18-20, 1090 Vienna, Austria; § Austrian Cluster for Tissue Regeneration, 1200 Vienna, Austria; ∥ Department of Physical Chemistry, University of Vienna, Währinger Str. 42, 1090 Vienna, Austria; ⊥ European Synchrotron Radiation Facility, 71 avenue des Martyrs, 38000 Grenoble, France; # Institute of Wood Technology and Renewable Materials, Department of Natural Sciences and Sustainable Resources, BOKU University, Konrad-Lorenz-Str. 24, 3430 Tulln an der Donau, Austria; g Department of Applied Science, William & Mary, Williamsburg, Virginia 23185, United States; ▽ Medical Systems Biophysics and Bioengineering, Leiden Academic Centre for Drug Research, Faculty of Science, Leiden University, 2333CC, Leiden, The Netherlands

**Keywords:** Material Characterization, Migratory Behavior, Peripheral Nerve Regeneration, Tubuliform silk, X-ray Scattering

## Abstract

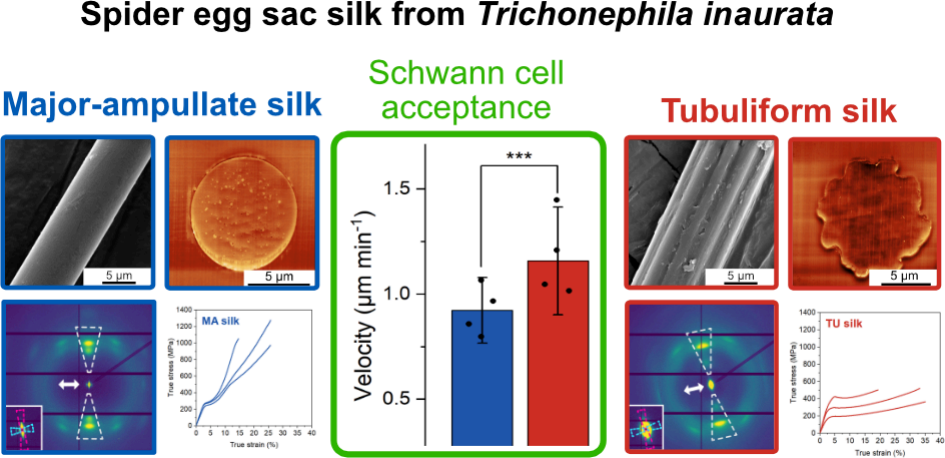

Spider silk (SPSI) is a promising candidate for use as a filler material in nerve guidance conduits (NGCs), facilitating peripheral nerve regeneration by providing a scaffold for Schwann cells (SCs) and axonal growth. However, the specific properties of SPSI that contribute to its regenerative success remain unclear. In this study, the egg sac silk of *Trichonephila (T.) inaurata* is investigated, which contains two distinct fiber types: tubuliform (TU) and major ampullate (MA) silk. These fibers serve as models to derive material parameters governing SC migration on natural silk substrates, since they are produced by the same spider, yet exhibiting distinct composition and morphology. In this paper, detailed characterization of the fibers’ material properties and *in vitro* evaluation of their SC-guiding performance were conducted. Live cell imaging revealed significantly enhanced SC mobility and directionality on TU silk compared to MA silk, which is remarkable, given the lack of studies on TU silk for nerve regeneration. Our results suggest that the distinct morphological and material properties of these fibers are critical to their nerve-guiding potential. These insights contribute to the optimization of NGC filler materials by identifying key parameters essential for effective nerve regeneration.

## 1 Introduction

Spider silk (SPSI) is a material long known for its biomedical applications. Already the ancient Greeks and Romans found an advantage in treating wounds with spider silk (SPSI) fibers.^1−3^ In 1710, the potential of SPSI for wound healing was already scientifically investigated.^4^ Since then, various studies on the possible biomedical uses of SPSI have been conducted, including suture material and tissue engineering.^5−9^ The application of SPSI as a luminal filler for nerve guidance conduits (NGCs) in nerve regeneration is an intriguing and promising area of research. While at the moment electroactive materials are frequently studied for this purpose,^10,11^ spider silk offers additional advantages, including excellent mechanical properties^12^ and fatigue behavior^13^ as well as no inorganic degradation byproducts. In this context, SPSI can provide directionality and thus serve as a guiding material for Schwann cells (SCs) and sprouting axons, enabling efficient regeneration.^14−19^ The silk type that has been the focus of previous studies for tissue engineering is silk from the major-ampullate (MA) gland, also known as dragline silk, from the genus *Trichonephila (T.)* (previously known as *Nephila*).^20^ In contrast, silk originating from spiders’ egg sacs has hardly been investigated for tissue engineering, with very few exceptions.^21,22^ One such study demonstrated the use of *T. edulis* egg sacs to engineer cartilage tissue.^22^ However, the properties of egg sac silk and its potential for nerve regeneration applications have not been explored so far.

Spiders’ egg sacs fulfill a crucial task in their reproductive biology, during which a female spider must protect her fertilized cocoons until the offspring hatch and mature into juvenile spiders. Therefore, she produces a tangled network of silk fibers, shielding her spiderlings against predators and environmental damage.^23,24^ Egg sacs are multilayered, complex structures that contain fibers from various glands,^25^ including major- and minor-ampullate, aciniform, pyriform and tubuliform glands. The composition of the egg sac varies considerably between species.^26^*T. clavata’s* egg sac is reported to consist of two different types of silk: Major-ampullate (MA) and tubuliform (TU) gland-derived silk.^27^ DNA libraries have been generated and amino acid analysis has been performed, indicating that tubuliform spidroin 1 (TuSp1) is the predominant component of the TU gland in all three species, which shows a lower glycine content, but an increased serine content compared to major-ampullate spidroins (MaSps).^28−30^

The TU silk of the spider *Argiope argentata* exhibits an irregular surface with knobs and grooves.^31^ Similarly, the TU silk of the spider *T. clavata* has been reported to exhibit longitudinal grooves and nodules on its surface.^27^ The mechanical performance of *T. clavipes* TU silk was initially investigated by Stauffer,^32^ who reported that it exhibited a brittle character. This finding contradicts the results of a more recent study on the behavior of *T. clavata* TU silk under tensile load, which demonstrated a low strength but high extensibility.^27^ Other species that have been studied for the mechanical performance of their TU silk include *Argiope argentata* and *Araneus diadematus*. The former was found to exhibit a high Young’s modulus and a low strength as well as a distinct yield region with a viscous postyield behavior.^31^ For *Araneus diadematus*, the tensile performance of dragline silk and TU silk was compared. TU silk exhibited similar maximum strain values but reduced strength relative to dragline silk. The observed differences in strength are attributed to the less compact packing of the β-sheets, which is caused by more voluminous amino acid side groups.^33−35^ In summary, except for the fiber mechanics, which has been studied for some species, the knowledge about TU silk properties is rather sporadic,^25^ and partially contradicting.^27,31,32^

Notably, egg sac silk has not been explored for its potential in nerve regeneration so far. The only reported observation is that SCs adhere to silk fibers derived from the egg sacs of *T. edulis*.^15^ The mobility of the SCs on egg sac silk fibers, however, which is essential for their nerve guidance ability, has not been studied. Furthermore, no distinction was made between MA silk and TU silk, although the two fibers–while both being present in the egg sac–exhibit different composition and morphology.

This is particularly intriguing, as previous studies have demonstrated that the velocity of SCs is strongly influenced by the substrate’s material properties, including stiffness,^36,37^ topography,^38^ primary protein structure and surface chemistry.^14,16^ In the present study, we take advantage of the two kinds of naturally spun fibers (MA silk and TU silk) coexisting next to each other in one egg sac deriving from different glands of the same animal. This provides us with a model system to investigate potential material attributes influencing SC migration, and thus medical applicability, while effectively reducing interindividual differences.

In this paper, we investigate the material properties of egg sac silks from *T. inaurata* and assess their acceptance by SCs. In live cell imaging experiments, we observe an even higher velocity of SCs on TU silk fibers compared to MA silk, thus suggesting that TU silk might outperform the conventional dragline silk. This is of particular value as dragline silk has been demonstrated to be highly suitable for guiding SCs and accelerating nerve regeneration.^17−19^ Extensive morphological, ultrastructural and mechanical characterization of the two fibers by scanning electron microscopy, Raman spectroscopy, synchrotron X-ray scattering, single fiber tensile testing and nanoindentation allows us to put this finding into context and provide an interpretation of the silk properties that influence the outcome of cell culture experiments. Given the restricted availability of the raw material spider silk and the natural variability, the long-term objective is to reproduce it artificially, for instance, through recombinant processes.^39−41^ Consequently, it is of paramount importance to ascertain which factors are crucial when designing it. Our work, therefore, offers significant inspiration for the future development of fiber-enhanced nerve guidance conduits to treat peripheral nerve injuries.

## 2 Materials and Methods

### 2.1 Harvesting of Silk

**Figure 1:**
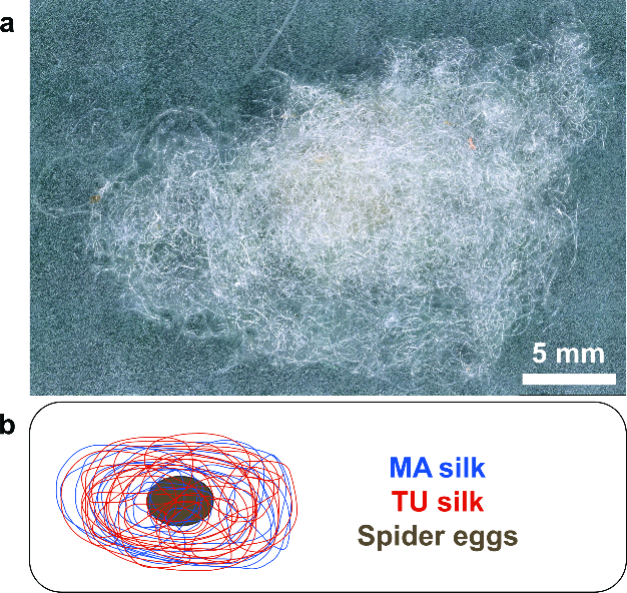
(a) Microscope image (Keyence VHX-5000) of sampled egg sac from *T. inaurata*. (b) Schematics of an egg sac showing random fiber distribution and location of spider egg agglomerate inside the entangled fibers.

*T. inaurata* spiders were housed under optimal temperature and humidity conditions.^42^ The spiders were fed twice a week with crickets (*Acheta domesticus*), and the webs were misted with water on a regular basis.^14^ Fresh, unfertilized egg sacs were collected from *T. inaurata* terraria. The egg sacs were thereby removed gently and were stored under ambient conditions with the exclusion of light until further usage. The egg sacs were all similar in size and silk fibers were loosely entangled around an agglomerate of spider eggs in the center (Figure 1). The two silk types, which were randomly distributed in the egg sac, were extracted manually and identified using a digital microscope (Keyence VHX-5000). For *T. clavata* TU and MA silk were reported as components of the egg sac.^27^ Because the silk is not forcibly extracted from the spiders but naturally spun, multiple gland types can be involved. Since wide-angle X-ray scattering data and morphological features (diameter, shape) fit previously studied forcibly silked MA silk we have a strong indication to believe, that the MA-like fiber we found in the egg sac of *T. inaurata* is silk derived from the MA gland (MA silk). We did not find other types of fibers, like aciniform and minor-ampullate silk, but this does not mean that they are not present in the egg sac of *T. inaurata*. The fibers might be just too thin. To prevent alteration of the material properties of the individual fibers great care was taken during the manipulation process to not prestretch them.

### 2.2 Morphological Characterization (SEM, AFM)

Scanning electron microscopy (SEM) was employed to examine the morphology of the SPSI types present in the egg sac of *T. inaurata*. Measurements were conducted on a FEI Quanta 250 FEG ESEM (Thermo Fisher Scientific) under high vacuum conditions and with an acceleration voltage of 10 kV. A bundle of fibers extracted from the egg sac as well as single, preseparated TU and MA silk fibers were mounted individually on SEM stubs using carbon tape as substrate. Subsequently, the samples were coated with an approximately 12 nm thick layer of gold via sputter coating (operated at 0.15 mbar of argon gas). Micrographs were recorded with the Everhart–Thornley detector in secondary electron mode to illustrate the characteristic morphology of representative silk samples.

Atomic force microscopy (AFM) measurements were performed on cross sections of silk fibers embedded in custom-mixed epoxy resin. The silk was aligned and clamped in flat embedding molds (Polysciences) and immersed in epoxy resin. The resin was then allowed to harden at 60 °C for 2 days. To check for the potential influence of epoxy embedding^43^ on the fibers’ mechanical or morphological properties, cryo-sections of SPSI embedded in optimal cutting temperature (OCT) compound were prepared (Figure S1). We found that the overall morphology of the fibers was similar to the shape of the cross sections from fibers embedded in epoxy. In addition, Raman measurements were performed on cross sections. The results showed that characteristic bands in the C–H region of the Raman spectra were similar for the cryo-embedded and resin-embedded samples, clearly differing from those of the pure resin (Figure S2), indicating that the possible penetration of epoxy in the silk does not have major effects on the spectra. Since we found similar results for morphology for both low- and high-temperature embedding procedures and no penetration of resin in the silk fiber, we assume that the mechanical and morphological properties are not altered significantly. Additionally, both silk types were embedded in epoxy by the same procedure allowing for relative comparison. The epoxy blocks were mounted on metal sample disks using a two-component epoxy adhesive (UHU-Endfest) and then cut perpendicular to the long axis of the fibers using an ultramicrotome (Leica RM 22 359) equipped with a histo-diamond knife (Diatome). Topography measurements were performed using a Dimension Icon AFM (Bruker) equipped with a ScanAsyst-Air silicon nitride probe (Bruker) with a nominal spring constant of 0.4 N m^–1^ and a nominal tip radius of 2 nm. The exact spring constants were obtained using the thermal tune method,^44^ and the deflection sensitivity was determined by recording a force curve on a sapphire sample. The scan rate was set to 0.3 Hz. Resolutions of 1024 × 1024 pixels, 256 × 256 pixels, and 128 × 128 pixels were chosen based on the scanned area. The scans were analyzed using the Gwyddion software (Version 2.61, GNU General Public License).^45^ A plane-level and a constant function were applied to remove the background. A second-order polynomial function was used in the vertical direction to remove cutting artifacts. To align the rows in the scanning direction a second-order polynomial function was applied in the horizontal direction.

### 2.3 Mechanical Performance (Nanoindentation, Tensile Testing)

Single SPSI fibers were tested for their tensile properties using a modified in situ nanoindenter used in tensile mode (ZHN/SEM Zwick/Roell) with two clamps to hold the ends of the 3D printed plastic support frame. The fibers were mounted on the frames using superglue. Care was taken to not prestress the fibers. The sides of the support frame were cut through with a soldering iron to obtain a free-hanging fiber. The motor of the nanoindenter was slightly moved together to verify that the fiber was not stressed and no force was applied. All specimens were tested in tension at a constant speed of 50 μm s^–1^, and force and displacement were recorded. To calculate stress and strain, the clamping length and diameter of each fiber were measured by digital microscopy (Keyence VHX-5000). The diameter was averaged from three positions, each with five measurements. The length was measured between the two glue points on the frame and was approximately 4 mm for all samples. Ten fibers from three individual egg sacs were measured leading to a total of 30 fibers per silk type.^46^ The measurements were performed at ambient temperature (24 °C) and at a relative humidity of 40%. The curve was fitted with a linear function from 0.2% to 1% strain to obtain Young’s modulus as a measure of the silk’s elastic properties. Yield strength was defined as the stress at 0.2% plastic strain.

For nanoindentation measurements, the same embedding procedure as for AFM measurements was performed. The blocks with embedded fibers were cut to a height of 8 mm using an Accutom-50 (Struers). To obtain a smooth surface transverse to the fibers’ long axis, the blocks were cut with a histo-diamond knife (Diatome). For measurements, the epoxy block was glued to 2 mm thick glass slides (Logitech) with a two-component epoxy glue (UHU Endfest). Nanoindentation measurements were performed on a Hysitron Triboindenter TI900 (Bruker). Ten individual fibers were selected for each silk type originating from one egg sac. Indentation was performed using a Berkovich-type diamond indenter in the center of the cross-section. A peak force of 100 μN was chosen to test the mechanical properties. The peak load was held for 20 s and an unloading speed of 33 μN s^–1^ was selected for the measurements. The obtained load–displacement curves were analyzed by the TriboScan Software using the Oliver–Pharr method^47,48^ resulting in values for the reduced modulus and the hardness. Since the derived reduced modulus for the silk fibers is 2 orders of magnitude smaller than the modulus of the indenter (approximately 7–11 GPa vs approximately 1140 GPa) the tip deformation can be neglected.

### 2.4 Secondary Protein Structure (Raman Spectroscopy)

Raman spectroscopy of single silk fibers fixed with superglue on a 3D-printed support was conducted with a WITec alpha 300A micro-Raman instrument and a 100*x*/0.9 objective. The spectra were generated with a frequency-doubled Nd:YAG laser (532 nm wavelength), an average power of 10 mW and an acquisition time of 5 min, and in backscatter geometry with a resolution of 2 cm^–1^. It was verified that the silk did not undergo any morphological changes during the measurements, by optical microscopy. Three spots on five fibers were measured for each silk type. The resulting spectra were corrected for fluorescent background. Spectra were recorded in xx, xz, zx, and zz orientations by means of two polarizers in the beam path to the silk and to the detector. The orientation insensitive spectra were calculated from these.^15,16,49,50^ By spectral decomposition of the conformation sensitive amide I regions and the side chains (1550–1750 cm^–1^), the secondary structure composition could be quantified.

### 2.5 Ultrastructural Investigations

Previous experiments have demonstrated the suitability of synchrotron radiation for studying individual SPSI fibers.^16,51−53^ X-ray nano diffraction measurements were performed in transmission mode at the nanofocus beamline (ID13) of the European Synchrotron Radiation Facility (ESRF). X-ray transparent Si_3_N_4_ membranes were used as a substrate for the SPSI fibers. MA and TU silk fibers were extracted from the egg sac of *T. inaurata* and fixated on the edge of the membranes using super glue. The monochromatic beam (λ = 0.08157 nm) was oriented perpendicular to the surface and focused to an approximately 0.1 × 0.1 μm^2^ full-width-half-maximum spot.

The sample-to-detector distance was set at 195 mm and 741 mm respectively, based on aluminum oxide (wide-angle X-ray scattering, WAXS) and silver behenate (small-angle X-ray scattering, SAXS) standards calibration. This means a low q limit due to the beamstop cutoff of 0.15 nm^–1^ in equatorial direction and 0.2 nm^–1^ in meridional direction. Scattering patterns covering the small- and wide-angle scattering range (SAXS/WAXS) were collected using an Eiger 4 M (Dectris) single photon counting pixel detector. Mesh scans were performed at room temperature (23 °C) in air at selected positions on the silk threads and sequences of scattering patterns were recorded. Silk fibers were scanned, with beam damage minimized by choosing a short exposure time of 10 ms for TU silk and 5 ms for thinner MA silk. Additionally, radiation damage propagation was reduced by choosing larger step sizes in the mesh in the direction of the fiber’s long axis.

The background data was selected from the sample displacement area and each background was subtracted from the corresponding measurement. Data reduction was performed using pyFAI, a Python library for azimuthal integration. More than 1000 scanning points were summed up inside the silk fiber to improve the signal-to-background ratio to achieve an average scattering pattern (Figure S3). Reflections were indexed according to the method reported in the literature.^54^ Bragg peaks and diffuse scattering (short-range order scattering - SRO) were fitted using Gaussians and nonlinear least-squares fitting using a Python code.

Lattice spacings (d_hkl_) are calculated from the scattering vector q, where d = q/2π. The particle size (L_hkl_) is derived from the full-width-at-half-maximum of the Gaussian-fitted Bragg peaks using Scherrer’s equation.^55^ The crystallinity parameter was calculated by dividing the total area under the Bragg peaks by the sum of the area under the Bragg peaks and diffuse scattering.

### 2.6 Isolation of Primary Rat Schwann Cells and Live Cell Imaging

Rat Schwann cells (rSCs) were isolated from sciatic nerves harvested from adult Lewis rats and cultured as described previously.^14^ All animals were sacrificed according to the Austrian Animal Testing Law (TVG 2012 § 2, 1. c) and Article 3 of the Directive 2010/63/EU of the European Parliament and of the Council on the Protection of Animals used for Scientific Purposes.^56^ Individual silk fibers of each type were mounted on 3D-printed silk holding frames (2 × 1 cm) by dissolving the top layer of the frame (polycarbonate) with dichloromethane (Sigma-Aldrich). Thirty silk fibers were fixed horizontally and vertically resulting in a crossed pattern in the center of the frame. Seeding of the rSCs on silk was performed according to Naghilou and Stadlmayr.^14,16^ In brief, the silk frames were sterilized by applying UV radiation for 40 min. An amount of 1×10^5^ rSCs were placed in a drop of 12.5 μL on the silk and kept at 37 °C and 5% CO_2_. After 1 h, the drop of cells and the silk on the frame were submerged in growth medium. The adhesion of rSCs after 1 h was documented by phase contrast microscopy (NIKON Eclipse Ts2R) with a 10*x*/0.25 objective. As evidenced in our preceding studies, the most notable differentiating factor between silk types is typically cell velocity. Only small discrepancies were identified in cell shape or proliferation.^14,16^ Consequently, our present investigation is concentrated on the measurement of cell velocities by performing live cell imaging experiments.

Live cell imaging started 2 h after seeding on an IX83 microscope (Olympus) using a 10*x*/0.3 objective. For a total of 15 h phase contrast images were recorded from multiple positions every 10 min using cellSens software (Version 3.2, Olympus). The random selection of positions also accounts for variations in spatial distribution and geometry, ensuring that the velocities remain comparable overall. Twenty cells per donor (*n* = 4) per silk type were randomly chosen and tracked. From those, at least 15 cells remained after removing outliers with a 0.05 Grubb test and disregarding the cells that migrated out of the imaging area during the 15 h. In total 68 and 72 cells for all four donors remained for TU and MA silk, respectively. Tracking was performed using the *Manual Tracking* plugin in the Fiji software (Version 2.9.0).^42^ A customized Mathematica (Version 12, Wolfram) evaluation algorithm was applied to correct frame movement during the measurement period. In detail, for each recorded image, the corresponding movement of the frame was tracked and subtracted from the cell movement, thus allowing for robust conclusions to be drawn. All samples were measured in a single run, thereby ensuring that all frames underwent the same instrumental movements. Migratory parameters such as total and Euclidean velocity as well as directness were calculated.

### 2.7 Statistical Methods

For tensile testing Raman spectroscopy measurements and *in vitro* experiments, mean average parameters and standard deviations as well as *p*-values were derived by a 2-way ANOVA (Origin 2021). Nanoindentation results were statistically analyzed by a 1-way ANOVA (Origin 2021). Biological and technical replicates per method are presented in Table 1. Ultrastructural parameters derived from SAXS and WAXS measurements were not analyzed statistically since only one or two fibers were measured per silk type due to time restrictions at synchrotron experiments. Nevertheless, data is derived from averaging over many single data points (for average data more than 5000) and can be interpreted by the standard deviations of the fits of the resulting scattering curves. A table in the Supporting Information provides an overview of all average measurement values and standard deviations (Table S1).

**Table 1:**
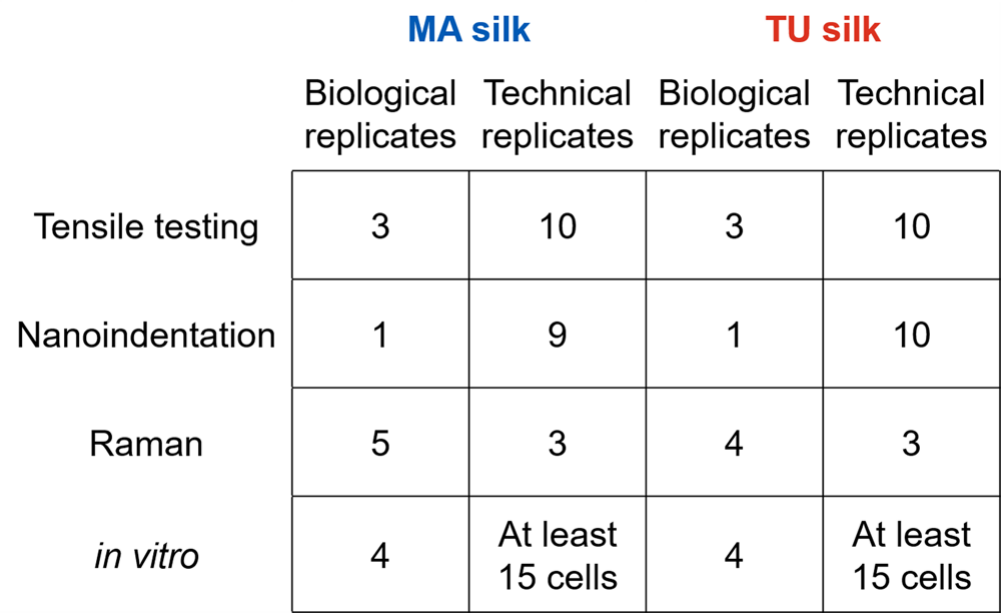
Biological and Technical Replicates for Each Silk Type for Different Measurement Methods

## 3 Results

### 3.1 Morphological Characteristics

**Figure 2:**
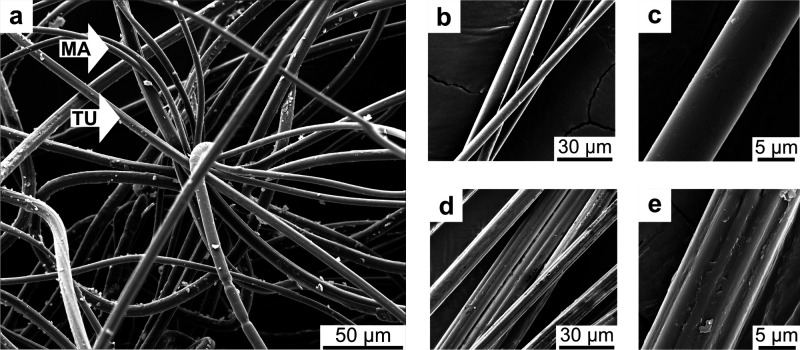
Scanning electron microscopy investigations on the egg sac of *T. inaurata*. (a) SEM micrograph showing entangled fibers. (b, c) MA silk fibers with a smooth appearance on the surface. (d, e) TU silk fibers with a longitudinally grooved surface.

SEM results reveal two distinctly differing morphologies of two fiber types that coexist in *T. inaurata* egg sacs (Figure 2 a). Overall, MA silk (Figure 2 b, c) has a smooth surface and a smaller fiber diameter compared to TU silk, which has longitudinal grooves on the surface and a larger fiber diameter (Figure 2 d, e). Diameter measurements from three different egg sacs, with ten fibers for each SPSI type, revealed a significant variation in fiber diameter with an average of 7.2 ± 1.2 μm for MA silk and 10.3 ± 1.2 μm for TU silk (Figure S4). We did not observe a layered structure of the egg sacs and the two types of SPSI were randomly distributed.

**Figure 3:**
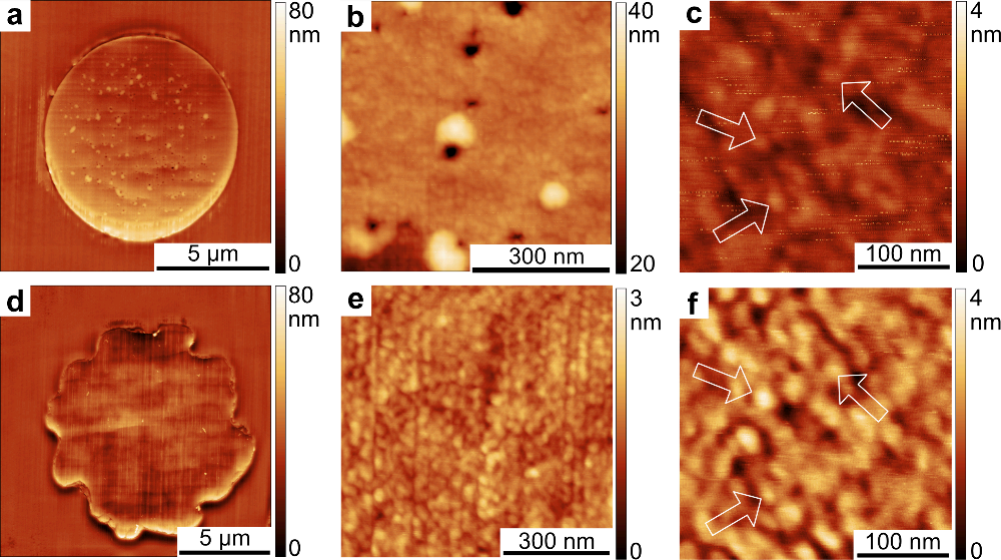
Atomic force microscopy topography measurements showing height channel from overview scans of cross sections of (a) MA silk and (d) TU silk. More detailed scan of (b) MA silk with cavities and (e) homogeneously structured TU silk. Highly magnified scans show the granular structure (arrows pointing to individual granules) of (c) MA silk and (f) TU silk. The cutting direction was vertical according to the displayed scans.

AFM was used to characterize the cross-section of the silk fibers morphologically. Besides the difference in fiber diameter, which was also shown by SEM, the shape of the fibers can be identified as almost perfectly round (MA silk, Figure 3 a) and irregularly shaped (TU silk, Figure 3 d). The number of grooves per TU fiber is approximately 13 ± 2. Their total depth is on average 875 ± 450 nm and their maximum width is 1.7 ± 0.5 μm, measured from ten different topography scans of the SPSI cross-section. Cavities smaller than 100 nm were found to be distributed throughout the cross-section of MA silk (Figure 3 a-c). In comparison, TU silk shows a more homogeneous topography where no such cavities were observed (Figure 3 d-f). Granular substructures with an average diameter of 20 to 30 nm were found on both silk types. Vertical cutting artifacts are visible due to the cutting process (Figure 3). Therefore, the size of the substructures was measured along these artifacts and not perpendicular.

### 3.2 Mechanical Behavior

**Figure 4:**
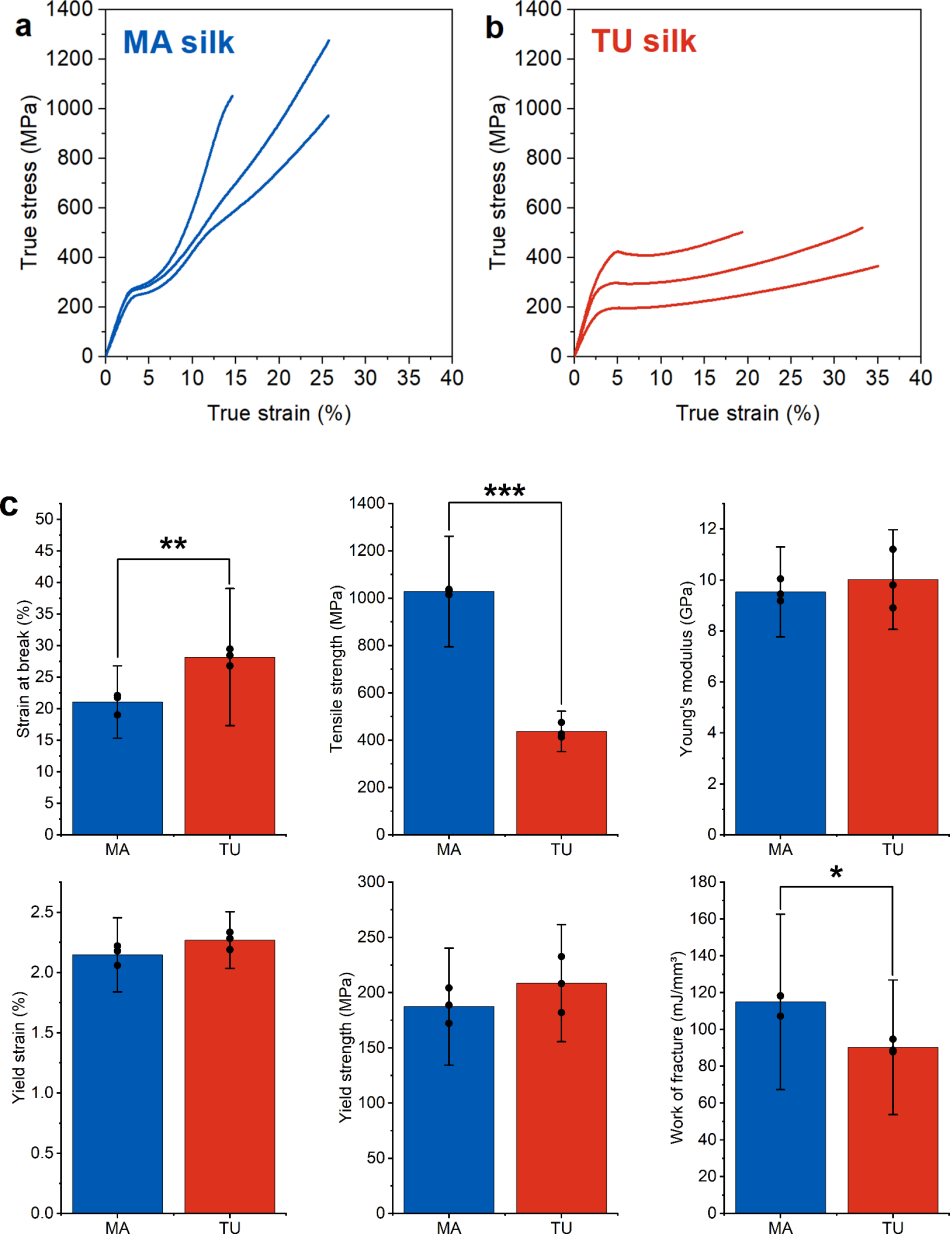
Mechanical performance in fiber long axis derived by single fiber tensile stretching experimental setup. (a) True stress vs. strain diagram with representative curves for MA silk and (b) for TU silk. (c) Tensile test parameters result from the shown stress–strain behavior with significant differences in maximum strength, strain and work of fracture. The bars with error bars display the average value for each silk type and its standard deviation. The individual points indicate the mean values per egg sac tested. (mean ± SD, *n* = 3) * *p* ≤ 0.05, ** *p* ≤ 0.01, *** *p* ≤ 0.001.

Single fiber tensile tests of TU and MA silk found in *T. inaurata* egg sacs revealed notable differences in mechanical performance between the two silk types, as evidenced by the true stress–strain curves (Figure 4, S4). The representation of tensile behavior using true stress and strain was chosen since we are measuring large deformations, whereas engineering stress and strain are small deformation approximations.^57,58^ Volume consistency while deforming the sample is an assumption needed to apply true stress and strain calculation. It was shown for MA silk from *Argiope trifasciata* that the fiber volume does not change upon stretching the fiber.^57^ However, to provide a complete image we also analyzed engineering stress and strain and provided the results which show similar behavior of the two silk types in their mechanical properties with respect to each other (Figure S7, S8, Table S1). Analyzing the tensile test data leads to significantly different extensibility between the two silk types, where TU silk shows a significantly higher strain at break compared to MA silk (Figure 4 c). By contrast, the maximum tensile strength and work of fracture are significantly higher for MA silk. All the other parameters evaluated, such as Young’s modulus, yield strain and yield strength, were found to be not significantly different for the two silk types (Figure 4). Nanoindentation experiments were performed to get a deeper understanding of the mechanical properties not only in the direction of the fiber long axis but in a mixed stress state composed of normal and shear stresses. The derived hardness and reduced modulus were found to be significantly higher for MA silk (Figure S5).

### 3.3 Ultrastructural Properties

#### 3.3.1 WAXS

**Figure 5:**
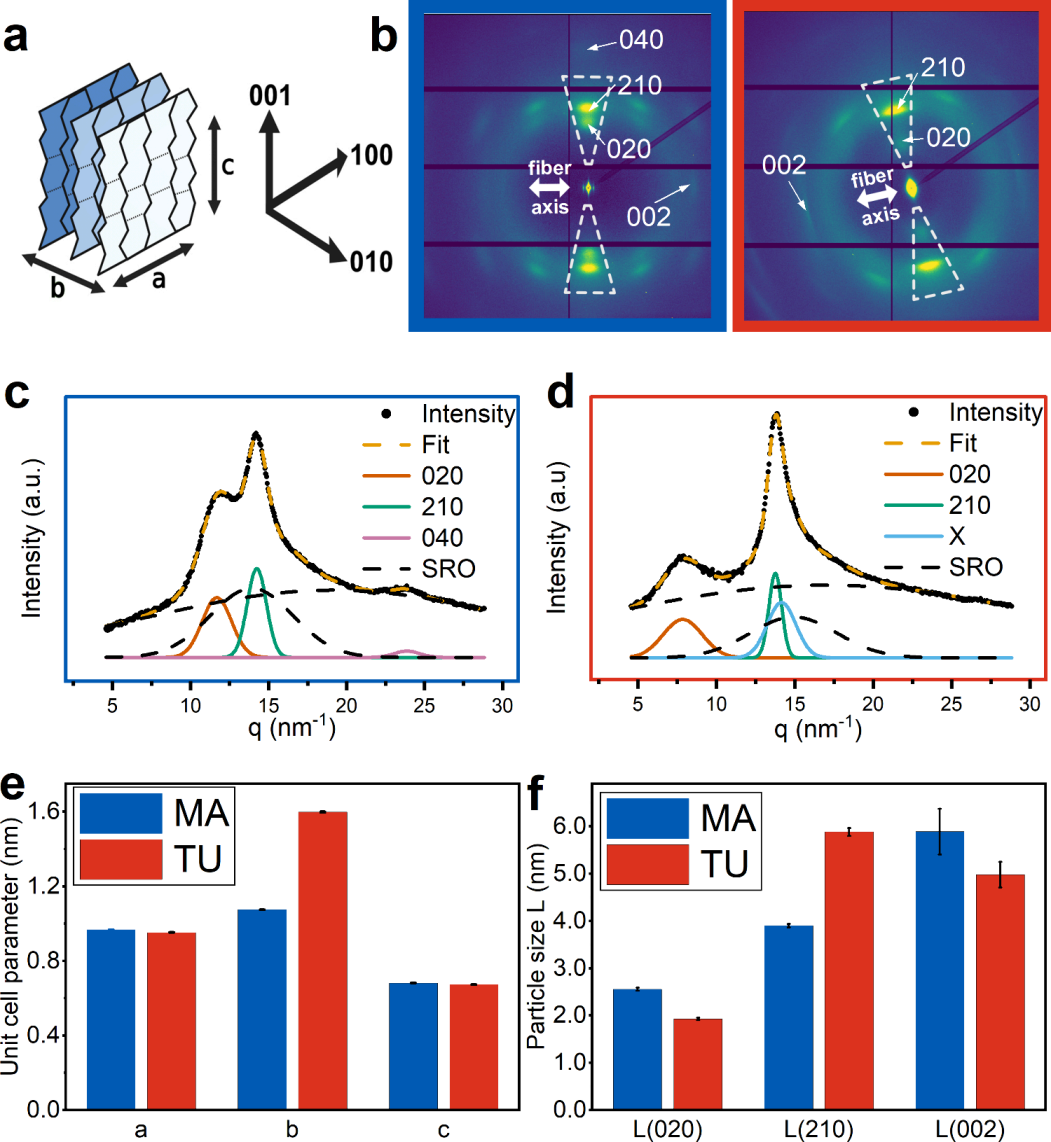
Ultrastructural investigations by nanobeam WAXS scanning of single silk fibers. (a) Directions in the lattice and schematic poly(L-alanine) nanocrystal β-sheet structure. The *c*-axis represents the fibers’ long axis. (b) Averaged scattering patterns for MA silk and TU silk. The double-sided arrows indicate the fiber’s long axis. The dashed triangle marks the area for integration resulting in 1d Intensity profiles for (c) MA silk and (d) TU silk. Bragg and SRO (Short-range-order) peaks were fitted by Gaussians. (e) Unit cell parameters a, b, and c of the orthorhombic unit cell for the two silk types. (f) Particle size derived by the Scherrer formula in different lattice directions for the two silk types. Error bars indicate the standard deviations originating from the fitting.

Ultrastructural investigations by nanobeam WAXS scanning of single silk fibers of each type revealed a clear difference in the peak position of the (020) Bragg peak (Figure 5 c, d). This results in a higher b-value for the orthorhombic unit cell (Figure 5 b)^59^ as well as an increased lattice parameter d_020_ (corresponding to the intersheet direction) for TU silk (Table S1). Furthermore, L_020_ and L_002_ are smaller and L_210_ is larger for TU silk compared to MA silk (Figure 5 f). The crystallinity parameter is similar for both silk types with average values of (0.11 ± 0.002) and (0.10 ± 0.002) for MA and TU silk, respectively. Fitting the pattern of TU silk required an additional Bragg peak as a Gaussian fit which is referred to as the x-peak (Figure 5 d). The (040) peak is not visible and thus is not fitted in the case of TU silk (Figure 5 c).

#### 3.3.2 SAXS

**Figure 6:**
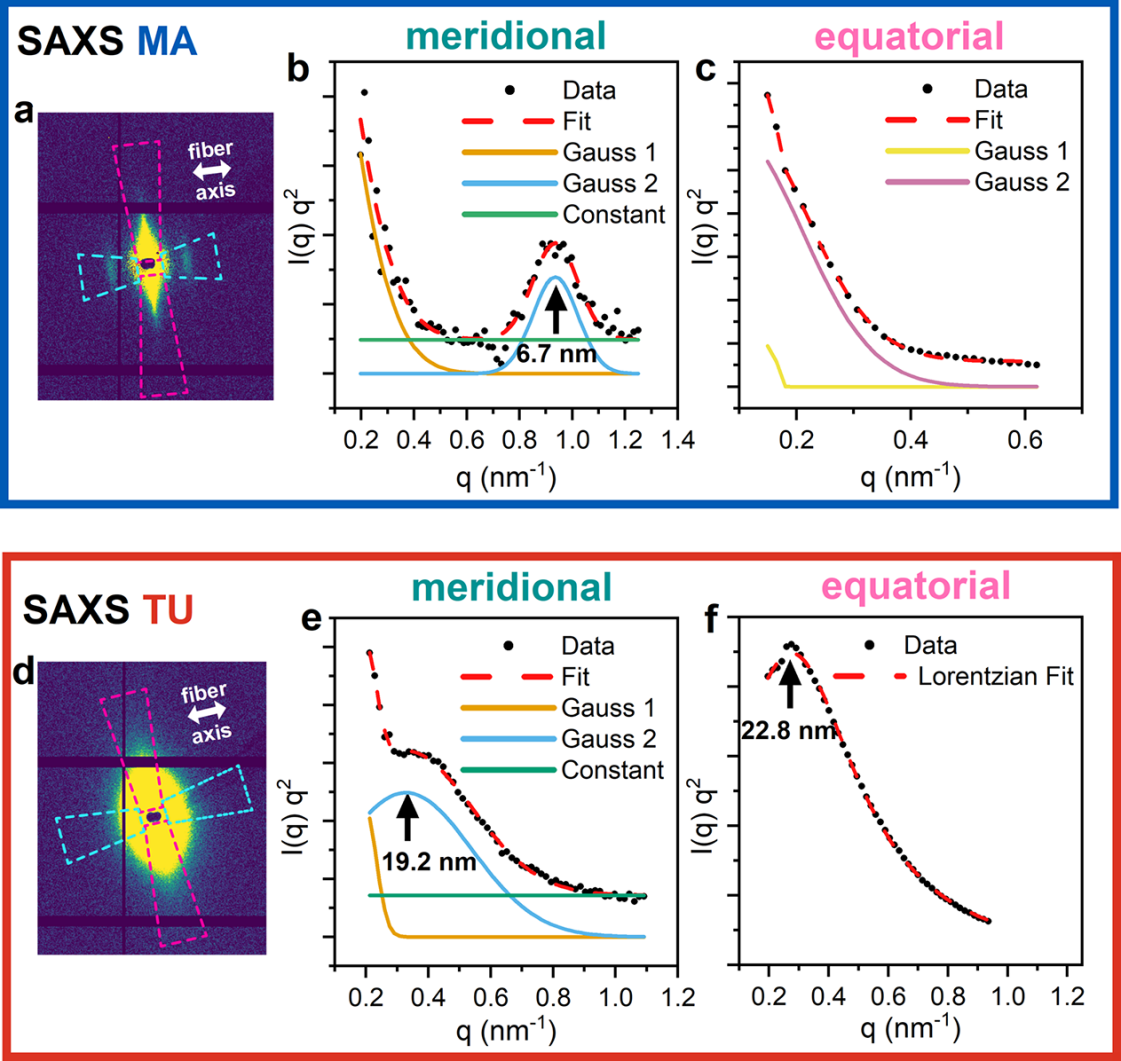
Ultrastructural investigations by nanobeam SAXS scanning of single silk fibers. (a) Average scattering pattern of MA silk. Magenta and cyan-colored sectors indicate meridional and equatorial directions, respectively. The double-sided arrow indicates the long axis of the fiber. Kratky plots (I(q)q^2^ vs q) of (b) meridional and (c) equatorial SAXS intensity fitted by Gaussians. (d) Average scattering pattern of TU silk. Magenta and cyan-colored sectors indicate meridional and equatorial directions, respectively. The double-sided arrow indicates the long axis of the fiber. Kratky plots (I(q)q^2^ vs q) of (e) meridional and (f) equatorial SAXS intensity fitted by Gaussians and Lorentzians.

SAXS curves from MA silk show a lamellar peak in the average meridional SAXS pattern of the fiber with a d-value of 6.7 nm (Figure 6 b). This refers to a structural feature in the long axis of the fiber. In the equatorial direction, no peak was found in the available q range (Figure 6 c). TU silk also shows a peak in the meridional SAXS profile corresponding to d = 19.2 nm (Figure 6 e) and a peak in the equatorial SAXS profile corresponding to d = 22.8 nm (Figure 6 f). The meridional d-value of TU silk is larger than the one for the lamellar SAXS peak in MA silk.

### 3.4 Secondary Structure Composition

**Figure 7:**
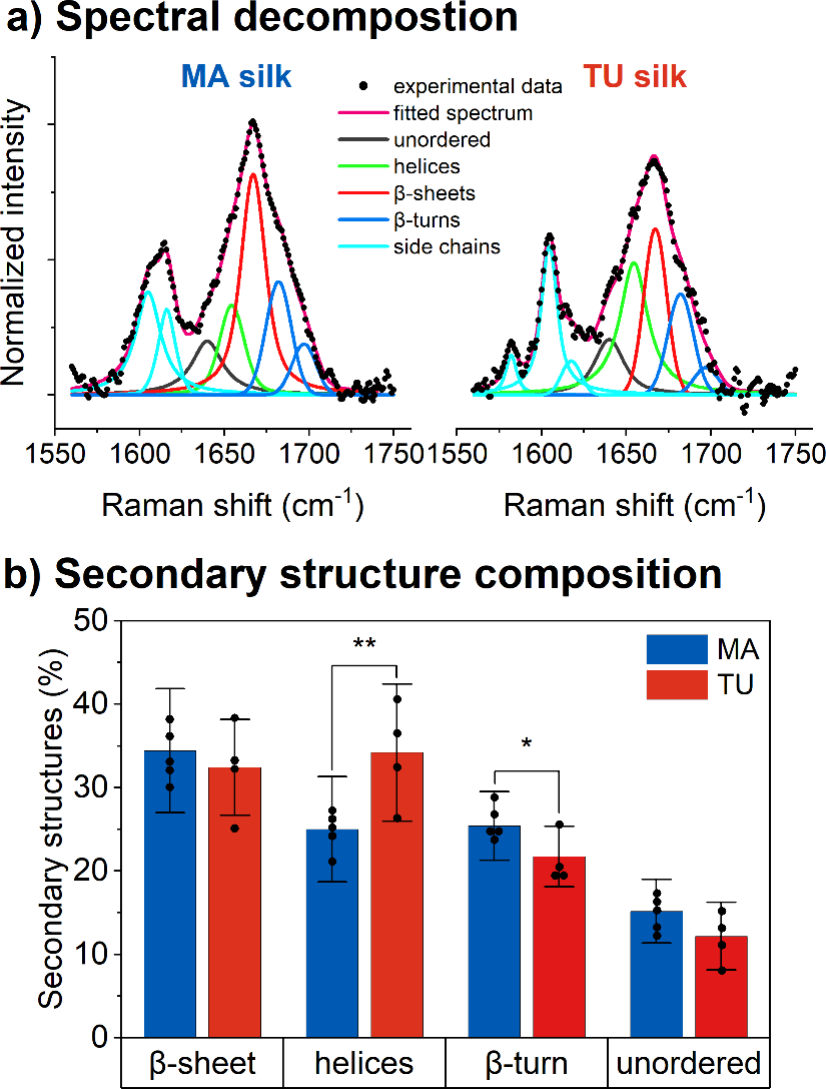
(a) Representative orientation insensitive Raman spectra with spectral decomposition of the amide I region for MA and TU silk. The spectra were normalized to least-squares deviation in the 1560–1750 cm^–1^ range for the MA silk. (b) Content of secondary protein structures calculated from the fit of orientation insensitive spectra. The bars with error bars display the average value for each silk type and its standard deviation. The individual points indicate the mean values per egg sac tested. (mean ± SD, *n* = 5 (MA), *n* = 4 (TU)) * *p* ≤ 0.05, ** *p* ≤ 0.01.

Raman spectroscopy was performed to obtain information on the secondary protein structure composition of the silk types found in the *T. inaurata* egg sacs. The orientation insensitive spectra for the amide I region (1560–1750 cm^–1^) were found to be similar for the two silk types (Figure 7 a). The bands at 1582 and 1604 cm^–1^ can be attributed to phenylalanine, which indicates a higher content of phenylalanine residues for TU silk.^30,49^

While the content of β-sheets was found to be insignificantly different between MA and TU silk, the content of helices was significantly higher in TU silk. The band components at 1682 and 1695 cm^–1^ arise from β-turns^49^ and were found to be significantly lower for TU silk compared to MA silk (Figure 7 b).

### 3.5 Schwann Cell Migration

**Figure 8:**
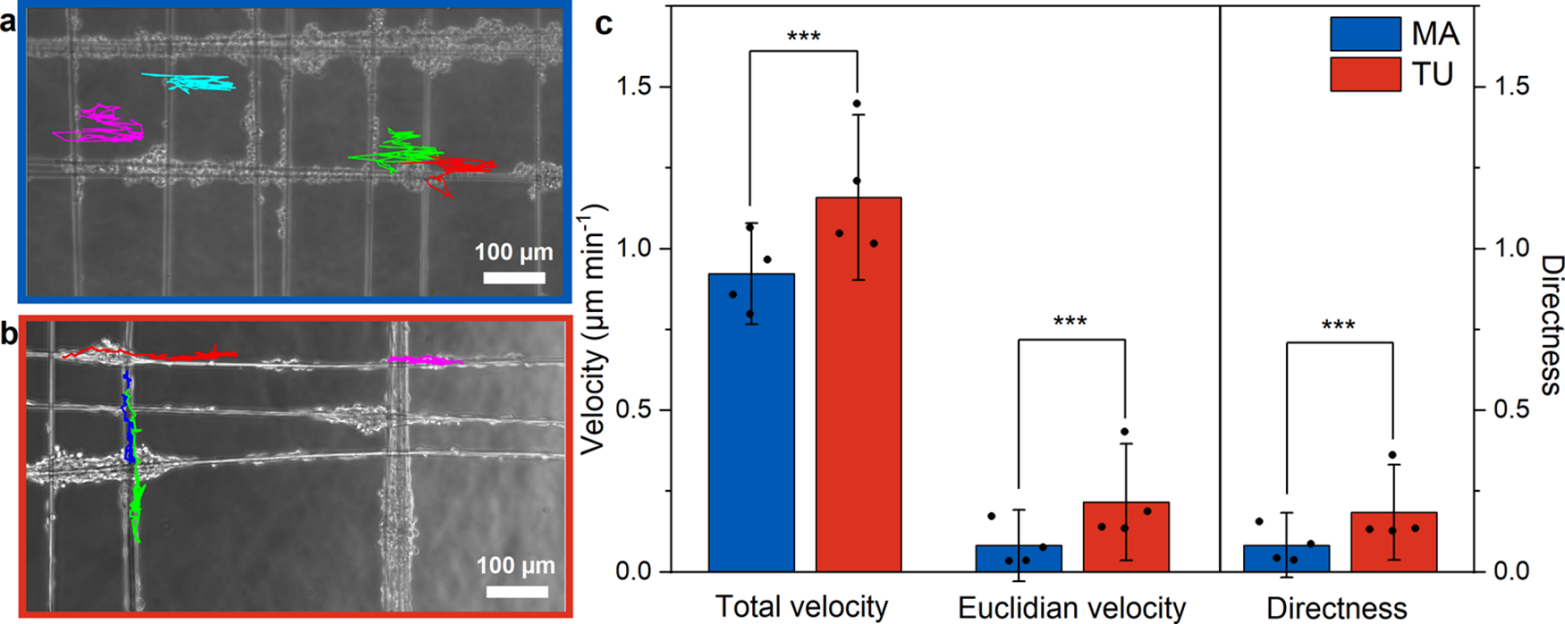
Representative phase contrast images of (a) MA and (b) TU silk with colored tracked cell paths. (c) Quantitative results from live cell imaging showing migratory potential and differences of rSCs on MA and TU silk fibers. Accumulated (total) velocity and effective (Euclidean) velocity in μm min^–1^ and directness. Dots per bar showing mean values for each donor. (mean ± SD, *n* = 4). *** *p* ≤0.001.

One important factor in assessing the medical applicability of a luminal filler for NGCs is the biomaterial’s capability to enable cell adhesion and migration of glial cells. The fast migration of SCs is of utmost importance as these cells should form directional tracks for the guidance of axonal sprouts, enabling the regrowing axons to reach the target organ as soon as possible to avoid chronic denervation.^60^ The mobility of rSCs on SPSI fibers extracted from the egg sac of *T. inaurata* was tested by live cell imaging to assess the possible regenerative potential of the fibers. Representative phase contrast images for both types of silk are displayed in Figure 8 (a, b) for MA and TU silk respectively, whereby the colored lines represent migration paths of individual rSCs. The movement of the frame was taken into account for the quantification of the parameters as described in section 2.6. All the assessed parameters, such as the total (accumulated) velocity, the Euclidean velocity as well as the directness were all found to be significantly higher on TU silk compared to MA silk (Figure 8 c), indicating the superior regenerative potential of TU silk in comparison to MA silk.

## 4 Discussion

In this study we investigated in detail the properties of two fibers found in the egg sac of *T. inaurata*. As both show quite different features, they serve as useful model systems to derive the parameters that govern the migratory potential of SCs on these fibers. The motility of SCs on guidance materials is an important factor in possible nerve regeneration applications. In particular, the TU silk studied here represents a promising type of SPSI, due to its superior SC guiding performance, together with a markedly different surface structure and mechanical properties as compared to the commonly used MA silk.

### 4.1 Material Properties

Particularly prominent is the different morphology of MA and TU silk found in the egg sac of *T. inaurata*. We found a significant difference in the diameter and the shape of the fiber’s cross-section. In literature, mostly cylindrical fiber morphologies have been investigated deriving from the major-ampullate gland.^61,62^ From the genus of *Trichonephila*, the TU silk of *T. clavata* is described with the features of longitudinal grooves and nodules on the surface. It is speculated that these grooves serve the spiderlings for better attachment.^27^ Some other TU silks, such as fibers from the egg sac of *Argiope aurantia* are also reported to show an irregular surface with knobs and grooves.

We found that TU silk exhibits higher extensibility and lower tensile strength compared to MA silk, whereas Young’s modulus was found to be not significantly different when tested in uniaxial conditions. Dragline and TU silk from *Araneus diadematus* were studied by Van Nimmen et al. and they summarize that these silks show similar strain, while TU silk exhibits lower strength and higher initial modulus. It is hypothesized that the lower strength of TU silk could be attributed to the larger side chain amino acids leading to a less compact structure of the nanocrystallites.^33−35^

Interestingly, our findings on the nanoindentation of the two different silks from the egg sac revealed a significant deviation in the reduced elastic modulus with a smaller value for TU silk. The reduced modulus from nanoindentation cannot be directly compared with the Young’s modulus derived from tensile tests. For once nanoindentation is performed in compression and not in tension. Also, the reduced modulus reflects a mixed stress state composed of normal and shear stresses. Therefore, to compare the two, the Poisson’s ratios from the different materials would be needed, which are not known. The reason for the lower values in the case of nanoindentation for TU silk can therefore be related to lower transversal modulus or shear modulus.^63^ This could be the result of weaker structures and interactions in the transverse direction to the fiber direction. For example, less densely packed β-sheets, which are predominantly aligned in the direction of the fibers, are one possible explanation.

In our study, the intersheet distance for TU silk was found to be significantly higher than for MA silk. The b-values of the unit cell are 1.08 nm for MA silk and 1.60 nm for TU silk. *T. clavipes* TU silk from the egg sac was studied by X-ray diffraction and the unit cell dimensions agree well with our values.^64^ Similar values are also reported for *T. senegalensis* egg sac silk.^54^ The large b-value of TU silk compared to MA silk is discussed as a result of larger side chain amino acids in the β-sheets such as valine, leucine, isoleucine, and phenylalanine.^27,28,54,64^ This is compatible with findings from mechanical testing, which show a lower strength and reduced modulus for TU silk.

In order to represent the full XRD curve, we included an additional Bragg peak at q = 14.14 nm^–1^ for TU silk. This peak is not present for MA silk and is not reported in the literature to date. The additional peak could be interpreted as a shifted (210) peak, potentially arising from regions within the silk where both, parallel and antiparallel β-sheets exist. While mostly antiparallel β-sheets are reported in silk, there are hints that mixtures could be present.^65^

The SAXS measurements revealed a lamellar peak in the meridional direction corresponding to a d = 6.7 nm for MA silk and 19.2 nm for TU silk. Similar *d*-spacings for MA silk ranging from 6.6 to 8 nm are reported for *A. bruennichi* and *T. clavata*.^66−68^ A long period of 10.5 nm in the transverse direction is reported for MA silk from *T. edulis*.^68^ In contrast to the literature, we did not measure an equatorial peak in that range for MA silk. For TU silk we found an equatorial peak related to a distance of 22.8 nm. This could reflect a possible microfibril diameter, since values from literature for MA silk cover a broad range from 10 to 100 nm and our AFM findings include circular structures in the range of 20 to 30 nm.^66,69,70^

In our Raman results, the orientation insensitive spectra for the amide I region were found to be similar for the two silk types and in agreement with the literature.^30,49^ Our results for the similar β-sheet content of the two silk types do fit the reported β-sheet content for *T. clavipes*. This is in line with our WAXS measurements, which did not reveal any significant difference in the crystallinity parameter for the two silk types. Similar to Rousseau et al. a significantly lower β-turn content can be attributed to TU silk compared to MA silk.^49^ The finding that the percentage of helices is significantly higher in TU silk than in MA silk can be one explanation for the higher extensibility of TU silk compared to MA silk. It has been reported that a higher content of α-helices in silk can lead to an increase in extensibility since the helices consist of easily movable chains.^20,71^

### 4.2 SC Response

Live cell imaging experiments on TU and MA silk from *T. inaurata* egg sac allowed us to observe a significantly higher total and effective velocity as well as directness for rSCs on TU silk compared to MA silk fibers. This is particularly remarkable since MA silk has been shown to be a very effective guiding material in various animal studies.^17−19^ The total velocity for rSCs on MA silk in our study fit the values for the total velocity reported for dragline silk from *T. inaurata*, whereas the effective velocity and directness found for MA silk in the present study were smaller than the ones reported.^14^ Another study on dragline silk of *T. edulis* also shows comparable values for the migratory potential of MA silk in this study.^16^ The performance of TU silk that we observe in cell culture is particularly intriguing when considering physiological parameters: The average reported axon regeneration rate after a peripheral nerve injury is about 1 mm per 24 h.^60^ The measured total velocity of SCs on TU silk (1.16 ± 0.26) μm min^–1^ is even above that rate.

It is well-known that cells react to structures of the substrate even smaller than the cell dimension.^72,73^ NGCs exhibiting a microstructure are playing a role as topographical cues and guidance.^74^ In particular, grooved NGCs are of interest to study nerve regeneration.^75−77^ In a study on films of recombinant SPSI, SCs have been shown to prefer the films with grooves over other patterns.^38^ It is known, that aligned orientations of fibers are preferred in regard to cell elongation and proliferation.^10,78,79^ Therefore, not only the NGCs themselves but also grooved aligned fibers as filler material are of interest, as it was shown that nanoscale grooves on electrospun microfibers (1–1.5 μm) enhanced the migration of SCs *in vitro*. The grooves’ optimal dimensions were approximately 24 nm in width and 30 nm in height.^80^*In vivo* experiments on rats also showed that electrospun, nanogrooved (200 nm groove width) nanofibers (500 nm diameter) with cellulose acetatebutyrate as filler material for NGCs considerably enhance peripheral nerve regeneration. One explanation for the enhanced migratory potential of nano- to microgrooved fibers might be an increase in the total fiber surface area, enhancing cell adhesion and proliferation^81^ as well as being a topographical cue guiding the cells.

Besides morphological features, among others, mechanical properties were shown to influence the migration of SCs on silk fibers.^14,16^ SCs are highly mechanosensitive and the stiffness of the extracellular matrix influences the behavior of SCs, like cell motility.^37,82^ A previous study comparing differently sterilized silk fibers from spider *T. edulis* revealed that silk fibers with higher Young’s modulus (autoclaved silk) show a lower velocity of SCs on the fibers.^16^ Stadlmayr et al. examined silk from different species and attributed the enhanced SC mobility of the jumping spider *Phidippus regius* to an interplay of primary protein structure and mechanical parameters, such as an increased hardness and reduced modulus.^14^ In our study, the higher SC velocity in TU silk was associated with a significantly lower hardness and reduced elastic modulus determined by nanoindentation, which is in contrast to previous findings,^14^ but confirms the multifactorial influence of material properties suggested by these authors.

Interestingly, the difference in the migratory potential of SCs on TU and MA silk in our study occurred although the tensile fiber stiffnesses were similar. Mechanical properties such as tensile strength and strain at break, which were significantly different for both types of silk, may not influence cell motility, since the mechanosensitive behavior of SCs is based on deformations much smaller than those measured when the fibers break. Mature SCs are surrounded by a basal lamina with an elastic modulus of 20–30 kPa, where in early stages of peripheral nervous system development the stiffness is estimated 1 kPa.^82^ Therefore, it is rather unlikely, that tensile strength and strain at break play a major role in influencing SC motility on the silk fibers. It is, however, conceivable that the reduced modulus as determined by nanoindentation is more representative of deformations during cell attachment than uniaxial tension. We found the reduced modulus to be smaller for TU silk compared to MA silk.

Further features of TU silk, like its higher content of α-helices might also result in a favorable acceptance of TU silk by SCs. Our results suggest that it is a combination of more than just one characteristic of TU silk resulting in an enhanced SC velocity. With this information, further steps can be taken to a targeted design of artificial silk fibers as a filler for NGCs by taking into account morphological features, secondary protein structure content, and mechanical performance as well as the proven advantages of SPSI for this purpose (excellent biocompatibility, biodegradability, cytocompatibility and minimal immunogenicity).^20^ For future research, a targeted design of regenerated SPSI fibers might bring even deeper insights into silk–cell interaction and improve the performance of NGCs.

## 5 Conclusion

In this study, we conducted an in-depth characterization and comparison in regard to SC acceptance of two types of silk from *T. inaurata* egg sacs: major ampullate (MA) silk and tubuliform (TU) silk. Our research yielded intriguing insights into the factors influencing SC migration, demonstrating the superiority of TU silk in this regard. Our results suggest that there are several features of the rarely studied TU silk playing a role in promoting SC movement on spider silk. The unique natural morphology of TU silk, characterized by its longitudinal grooves, the mechanical characteristics as well as the secondary protein structure content have been identified as possible influencing factors. This discovery is particularly exciting because it is the first time that TU silk has been studied in regard to nerve regeneration and was shown to enhance cell movement and even outperforms previously studied material. This is in particular promising, since already different natural silk fibers have been shown to be a very effective guiding material for SCs.^17−19^ Therefore, this performance might be even improved by a targeted production of artificial silk fibers taking the special properties of TU silk into account. Artificially produced fibers can mitigate the disadvantages of using natural silk due to its limited availability and natural variability. The implications of our research are significant, opening up new avenues for utilizing silk fibers or recombinant analogues in biomedical applications.

## Data Availability

Data is provided online under DOI: 10.5281/zenodo.14283026.

## Supporting Information Available

Detailed SAXS maps of MA and TU silk. Diameters of MA and TU silk for tensile testing. Results and representative curves from Nanoindentation experiments. All curves from tensile testing. Tensile testing data and all curves evaluated applying engineering strain and stress. A Table showing average values and standard deviations for the reported experiments (PDF)

## Abbreviations

MA: Major-ampullate
MaSps: Major-ampullate spidroins
NGCs: Nerve guidance conduits
SAXS: Small-angle X-ray scattering
SCs: Schwann cells
SPSI: Spider silk
SRO: Short-range order
*T.*: *Trichonephila*
TU: Tubuliform
TuSp1: Tubuliform spidroin 1
WAXS: Wide-angle X-ray scattering
