# Supplementary material for: Exploring the Unique Properties and Superior Schwann Cell Guiding Abilities of Spider Egg Sac Silk
Source: ACS Appl Bio Mater. 2025 Jan 17;8(2):1307–19. doi: 10.1021/acsabm.4c01587 (PMC11836930; doi:10.1021/acsabm.4c01587)
Supplement: Supplementary file 1 — mt4c01587_si_001.pdf [file mt4c01587_si_001.pdf]

Supplementary Information

Exploring the Unique Properties and Superior Schwann Cell Guiding Abilities of Spider Egg Sac Silk

Karolina Peter [a], Sarah Stadlmayr [b,c], Aida Naghilou [b,c], Leon Ploszczanski [a], Manuel Hofmann [d], Christian Riekel [e], Jiliang Liu [e], Manfred Burghammer [e], Claudia Gusenbauer [f], Johannes Konnerth [f], Hannes C. Schniepp [g], Harald Rennhofer [a], Gerhard Sinn [a], Christine Radtke [b,c], Helga C. Lichtenegger [a*]

[a] Institute for Physics and Materials Science, Department of Material Sciences and Process Engineering, BOKU University, Peter-Jordan-Str. 82, 1190 Vienna, Austria
[b] Department of Plastic, Reconstructive and Aesthetic Surgery, Medical University of Vienna, Währinger Gürtel 18-20, 1090 Vienna, Austria
[c] Austrian Cluster for Tissue Regeneration, Vienna, Austria
[d] Department of Physical Chemistry, University of Vienna, Währinger Str. 42, 1090 Vienna, Austria
[e] European Synchrotron Radiation Facility, 71 avenue des Martyrs, 38000 Grenoble, France
[f] Institute of Wood Technology and Renewable Materials, Department of Material Sciences and Process Engineering, University of Natural Resources and Life Sciences, Konrad-Lorenz-Str. 24, 3430 Tulln an der Donau, Austria
[g] Department of Applied Science, William & Mary, Williamsburg, VA 23185, United States

* E-Mail: helga.lichtenegger@boku.ac.at

Pages: 7

Figures: 8

Tables: 1

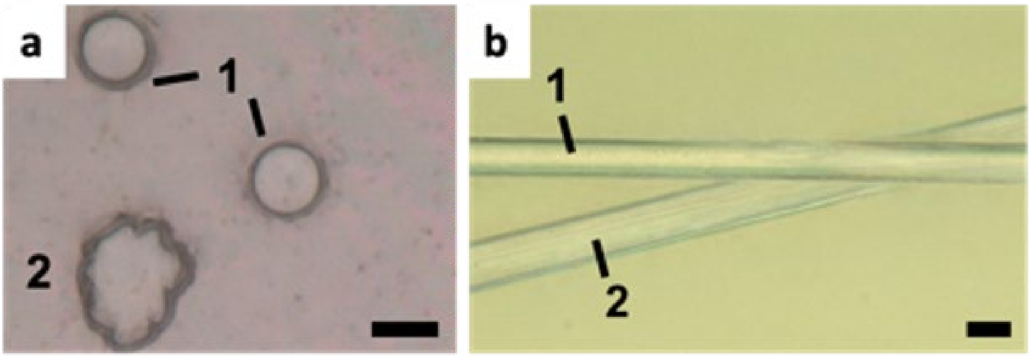

Figure S1 Microscope images (Keyence VHX-5000) of (a) 3 µm thick cryo-section and (b) of native fibers with of (1) MA and (2) TU silk from the egg sac of *T. inaurata*. Scale bars are 10 µm each.

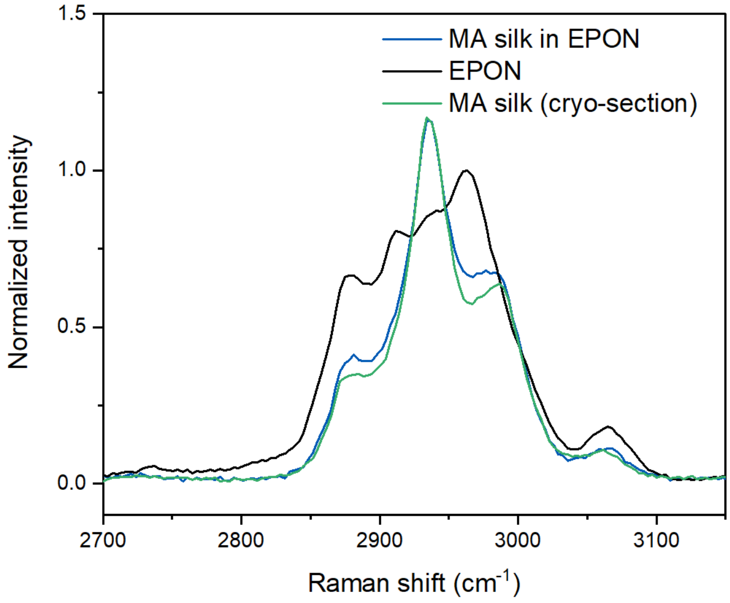

Figure S2 Raman intensity spectra of MA silk from cross-sections of *T. inaurata* showing the C-H stretching region. Blue: Embedded in Epon. Green: Cryo-section in OCT (Optimal cutting temperature compound). Black: Pure Epon resin.

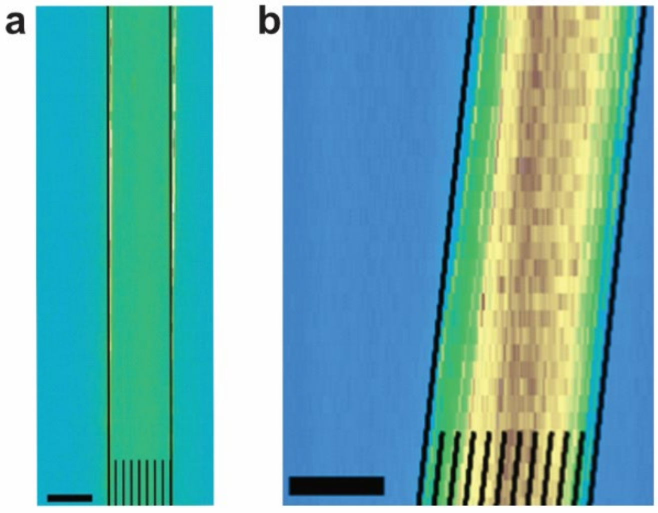

Figure S3 SAXS maps of (a) TU and (b) MA silk and the regions of interest (ROIs) for data analysis. Scale bars are 5 μm.

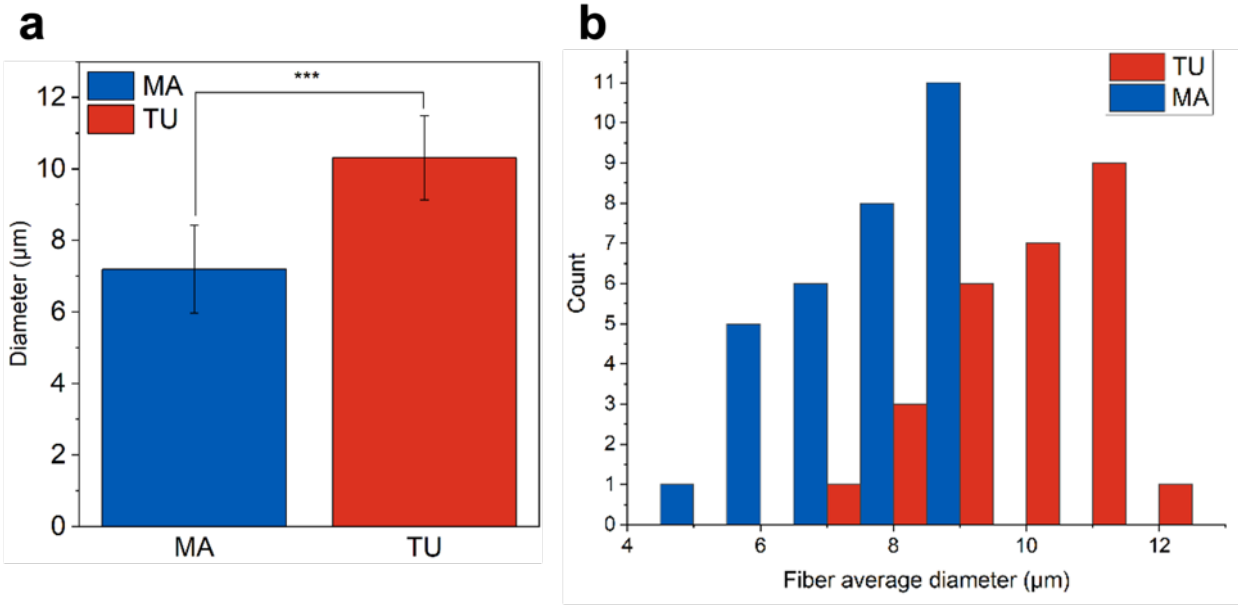

Figure S4 a) Average diameters (± SD) of major-ampullate (MA) and tubuliform (TU) silk used for tensile testing. *** p ≤ 0.001. b) Histogram displaying fiber diameter size distribution for MA and TU silk.

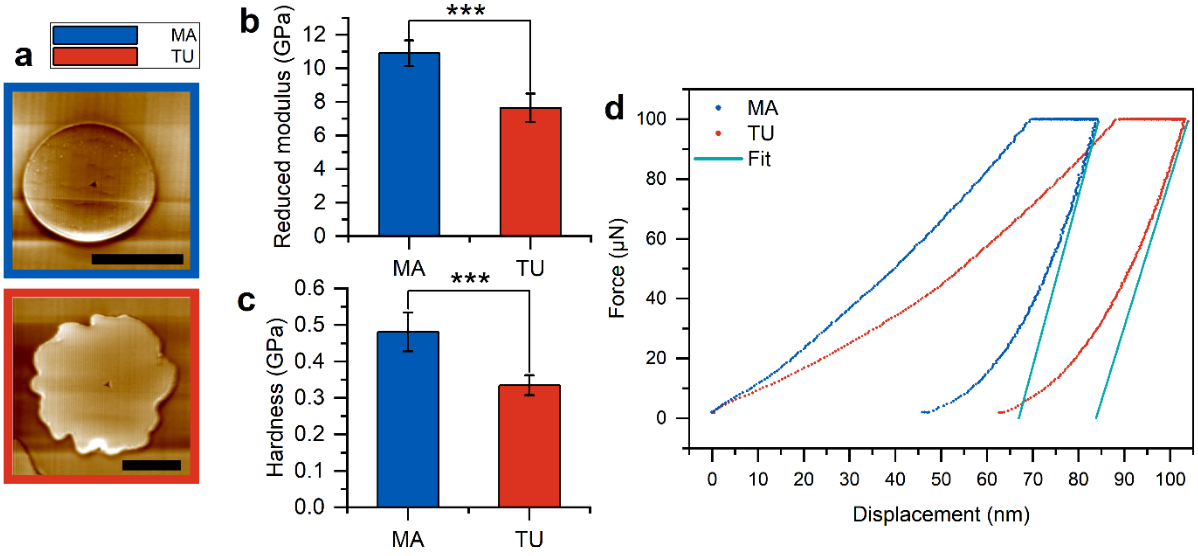

Figure S5 Nanoindentation experimental results. (a) Topography scans after performing an indent on tubuliform silk and major-ampullate silk. Scale bars are 5 μm each. (b) Reduced modulus and (c) hardness derived from the indentation of 10 cross sections for each silk type showing significantly higher values for MA silk. (mean ± SD, n=1) *** p ≤ 0.001 (d) Representative force vs. displacement curves for both silk types with fitted slopes.

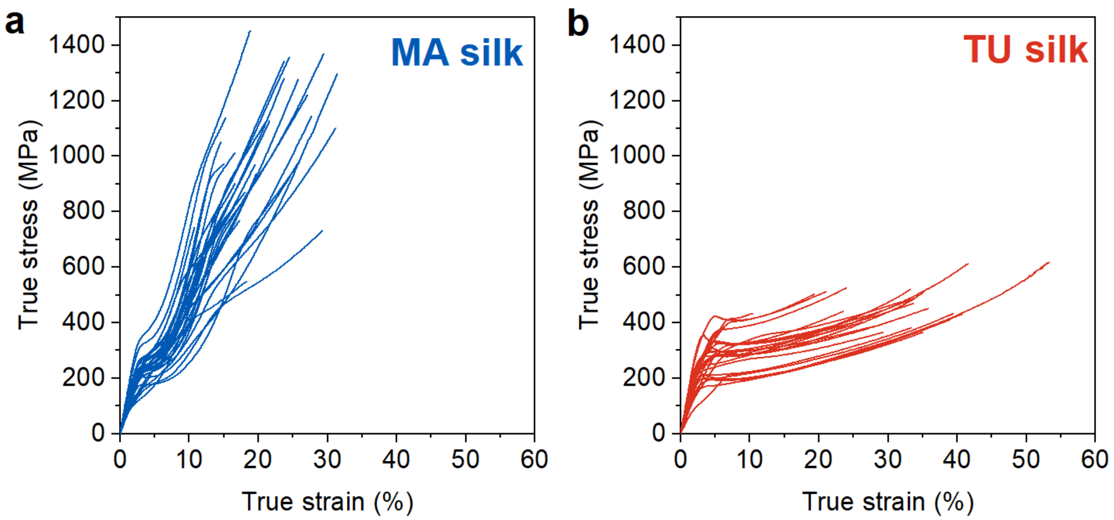

Figure S6 All measured true stress vs. true strain curves for (a) MA and (b) TU silk.

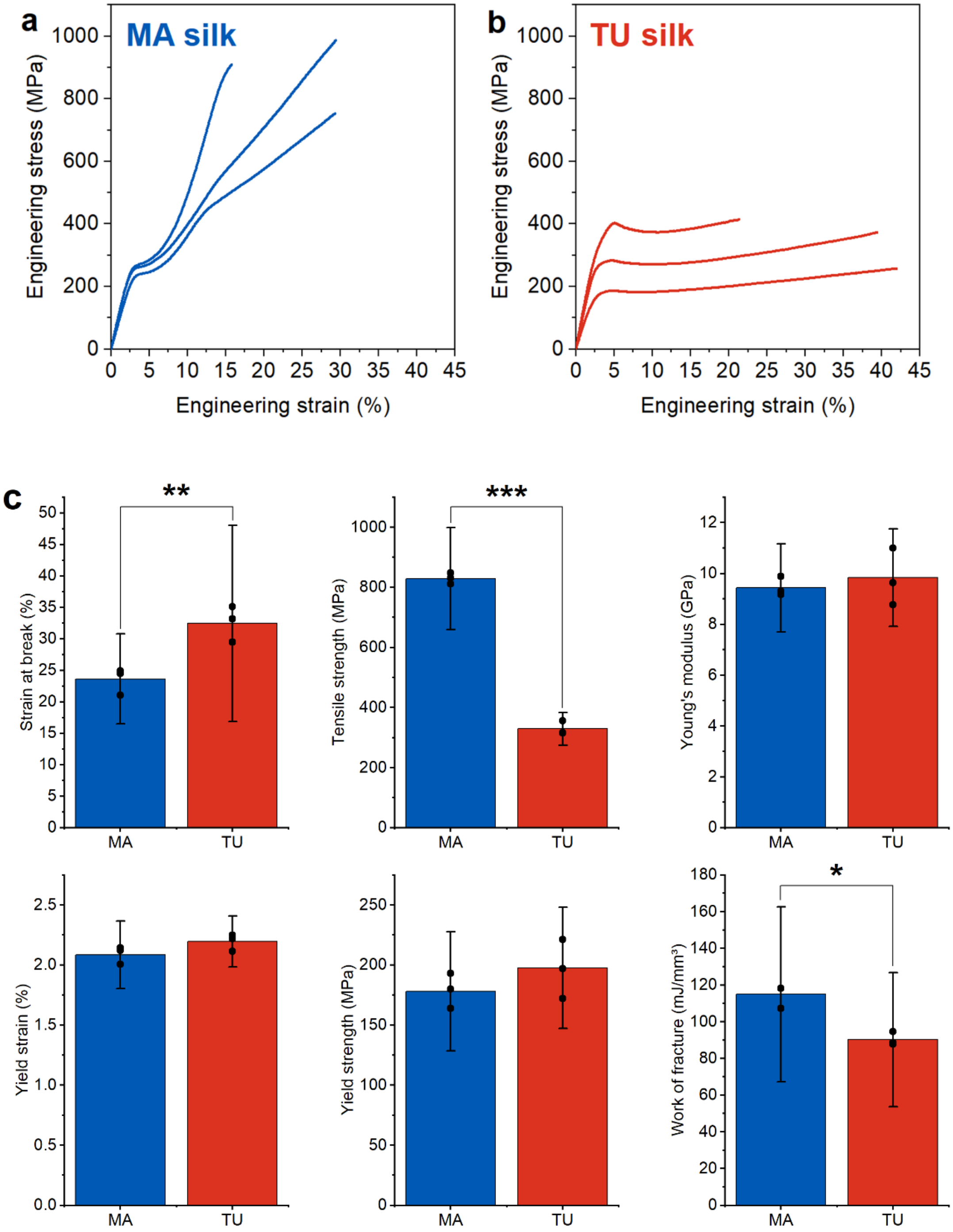

Figure S7 Mechanical performance in fiber long axis derived by single fiber tensile stretching experimental setup. (a) Engineering stress vs. engineering strain diagram with representative curves for MA silk and (b) for TU silk. (c) Tensile test parameters result from the shown stress-strain behavior with significant differences in tensile strength, strain at break and work of fracture. The bars with error bars display the average value for each silk type and its standard deviation. The individual points indicate the mean values per egg sac tested. (mean ± SD, $n = 3$) * $p \leq 0.05$, ** $p \leq 0.01$, *** $p \leq 0.001$.

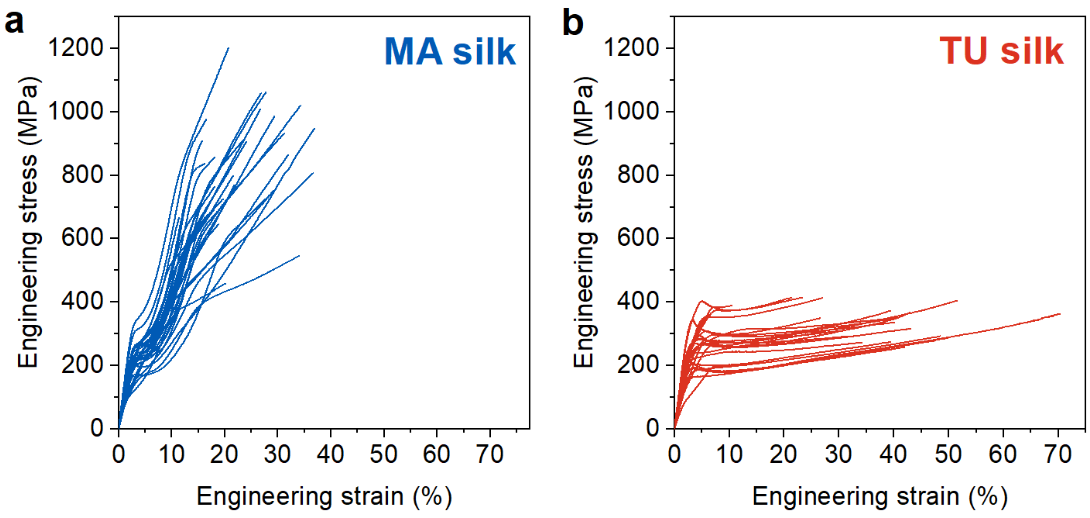

Figure S8 All measured engineering stress vs. engineering strain curves for (a) MA and (b) TU silk.

Table S1 Overview of average values and standard deviation per method for MA and TU silk.

**Average values ± SD**		
**Tensile tests (true)**	**MA**	**TU**
Strain at break (%)	21.05 ± 5.75	28.17 ± 10.85
Tensile strength (MPa)	1027.98 ± 234.02	437.38 ± 85.42
Young's modulus (GPa)	9.53 ± 1.77	10.02 ± 1.95
Yield strain (%)	2.15 ± 0.31	2.27 ± 0.23
Yield strength (MPa)	187.28 ± 52.99	208.43 ± 52.99
Work of fracure (mJ/mm^3)	114.94 ± 47.71	90.34 ± 36.56
**Tensile tests (engineering)**	**MA**	**TU**
Strain at break (%)	23.62 ± 7.16	32.47 ± 15.59
Tensile strength (MPa)	828.92 ± 169.27	329.10 ± 53.99
Young's modulus (GPa)	9.44 ± 1.73	9.84 ± 1.92
Yield strain (%)	2.09 ± 0.28	2.20 ± 0.21
Yield strength (MPa)	178.08 ± 49.64	197.66 ± 50.39
Work of fracure (mJ/mm^3)	114.96 ± 47.72	90.34 ± 36.56

**Average values ± SD in nm**		
**WAXS**	**MA**	**TU**
a	0.9672 ± 0.0003	0.9530 ± 0.0003
b	1.0705 ± 0.0008	1.5989 ± 0.0026
c	0.6813 ±0.0010	0.6736 ± 0.0007
L_{020}	2.5561 ± 0.0323	1.9302 ± 0.0272
L_{210}	3.9003 ± 0.0354	5.8822 ± 0.0822
L_{002}	5.8891 ± 0.4835	4.9805 ± 0.2698
d_{020}	0.5375 ± 0.0004	0.7995 ± 0.0013

d_{210}	0.4410 ± 0.0001	0.4566 ± 0.0001
d_{002}	0.3406 ± 0.0005	0.3368 ± 0.0004

Average values ± SD in nm^-1		
**SAXS**	**MA**	**TU**
q (meridional gaussian 2)	6.71 ± 0.04	19.16 ± 0.47
q (equatorial Lorentzian)	-	22.78 ± 0.09

Average values ± SD in %		
**Raman**	**MA**	**TU**
β-sheet	31.39 ± 2.93	33.26 ± 4.43
helices	24.12 ± 2.99	34.32 ± 6.12
β-turn	28.48 ± 1.71	21.75 ± 3.28
unordered	16.01 ± 4.27	10.67 ± 3.51

Average values ± SD		
**LCI**	**MA**	**TU**
Total velocity (μm/min)	0.92 ± 0.16	1.16 ± 0.26
Euclidean velocity (μm/min)	0.08 ± 0.11	0.22 ± 0.18
Directness (a.u.)	0.08 ± 0.10	0.18 ± 0.15
